# Thyrotoxicosis-Induced Cardiomyopathy With Systolic Dysfunction

**DOI:** 10.7759/cureus.33988

**Published:** 2023-01-20

**Authors:** Yusuf Khalil, Michael D Dube, Larry Woods

**Affiliations:** 1 Medicine, Northeast Ohio Medical University, Rootstown, USA; 2 Cardiology, Trumbull Regional Medical Center, Warren, USA

**Keywords:** congestive heart failure, dilated cardiomyopathy, hyperthyroidism, systolic dysfunction, thyrotoxicosis

## Abstract

Thyrotoxicosis-induced dilated cardiomyopathy is a rare but potentially life-threatening complication of thyrotoxicosis, with an incidence of <1%. This condition is characterized by a dilatation of the ventricular chamber and a decrease in cardiac contractility. Untreated, it can lead to irreversible changes in cardiac structure and function, including dilated ventricular chamber, a decrease in ejection fraction (EF), and an increased risk of atrial fibrillation.

We present a case of a 39-year-old patient with a diagnosis of thyrotoxicosis-induced acute heart failure. A two-dimensional (2D) echocardiogram disclosed an ejection fraction of 36%, with diffuse mild dilation of the atria and ventricles with trace mitral and tricuspid regurgitation. The anti-thyroid-stimulating hormone (TSH) receptor was positive, and Grave’s disease was diagnosed. The patient eventually returned to baseline functional status and could return to basic activities of daily living without limitations. The patient was encouraged to follow up with outpatient cardiology.

Early diagnosis of cardiac involvement in patients with thyrotoxicosis is critical. Promptly delivered intensive treatment with the rapid achievement of euthyroid state can potentially reverse cardiac dysfunction and improve patient outcomes.

## Introduction

Thyrotoxicosis is a clinical manifestation of excess thyroid hormone action at the tissue level secondary to inappropriately high circulating hormone concentrations [1]. Hyperthyroidism is a condition that may predispose a patient to thyrotoxicosis due to excess thyroid hormone synthesis. Thyrotoxicosis, as well as hyperthyroidism, are well-known causes of left ventricular (LV) hypertrophy and congestive heart failure (CHF) [2-5]. Thyroid hormone causes increased expression of myocardial sarcoplasmic reticulum calcium-dependent adenosine triphosphate (ATP), which increases heart rate and myocardial contractility with a resultant increase in cardiac output. Thyroid hormone also decreases systemic vascular resistance (SVR) and afterload through arterial smooth muscle relaxation. The decrease in SVR leads to the activation of the renin-angiotensin-aldosterone system (RAAS), which increases sodium reabsorption and expands blood volume to increase preload [6]. Therefore, a sustained cardiac output and preload increase may lead to cardiac failure.

There is a vast amount of literature on the relationship between thyrotoxicosis as well as hyperthyroidism and high-output heart failure and left ventricular hypertrophy [7-13]. However, there has been a paucity of articles discussing the development of severe left ventricular dysfunction in a patient with acute thyrotoxicosis [14,15]. Dahl et al. found that although 6% of thyrotoxic individuals developed symptoms of heart failure, less than 1% developed dilated cardiomyopathy with impaired left ventricular systolic function [16]. Additionally, case reports on the development of dilated cardiomyopathy with reduced ejection fraction (EF) secondary to thyrotoxicosis have been in middle-aged or elderly patients [14,15]. We present a novel case of a young patient who presented with thyrotoxicosis and subsequently developed dilated cardiomyopathy with reduced ejection fraction and global hypokinesis.

## Case presentation

This patient is a 39-year-old African American male with a past medical history of tobacco and cannabinoid use, fentanyl abuse, and asthma who presented to the emergency department with an abrupt onset of shortness of breath and palpitations. The patient’s symptoms began the evening prior. The patient was experiencing chest pain on the left side, which is accompanied by neck pain. The chest pain and dyspnea (shortness of breath) appeared to be worse when the patient was walking upstairs. It is not specified whether the pain is radiating or not. His last fentanyl use was one month prior. At the time of admission, he had been in a rehabilitation facility for one month.

He denied fever, chills, recent weight loss, change in vision, nausea, vomiting, headache, diarrhea, recent sick contacts, or previous episodes of his presenting symptoms.

The physical examination revealed an afebrile patient, with a blood pressure of 131/85 mmHg, tachycardic (110 bpm), and tachypneic (22 breaths/minute), with an oxygen saturation of 100% on room air. Findings included shortness of breath with lung fields clear to auscultation bilaterally and hand tremors on outstretched extremities bilaterally. The remaining physical examination results were unremarkable.

**Figure 1:**
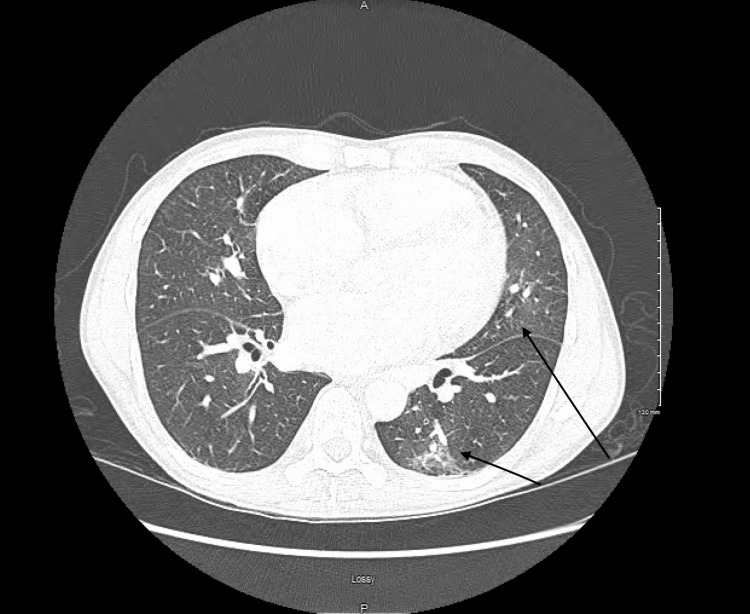
CTPA of the chest showing no signs of pulmonary embolism, patchy ground glass opacities within the lingula and left lower lobe, mild dependent changes with subsegmental atelectasis within the bilateral lower lobes, and mildly enlarged main pulmonary artery (black arrows) CTPA: computed tomography pulmonary angiogram

**Figure 2:**
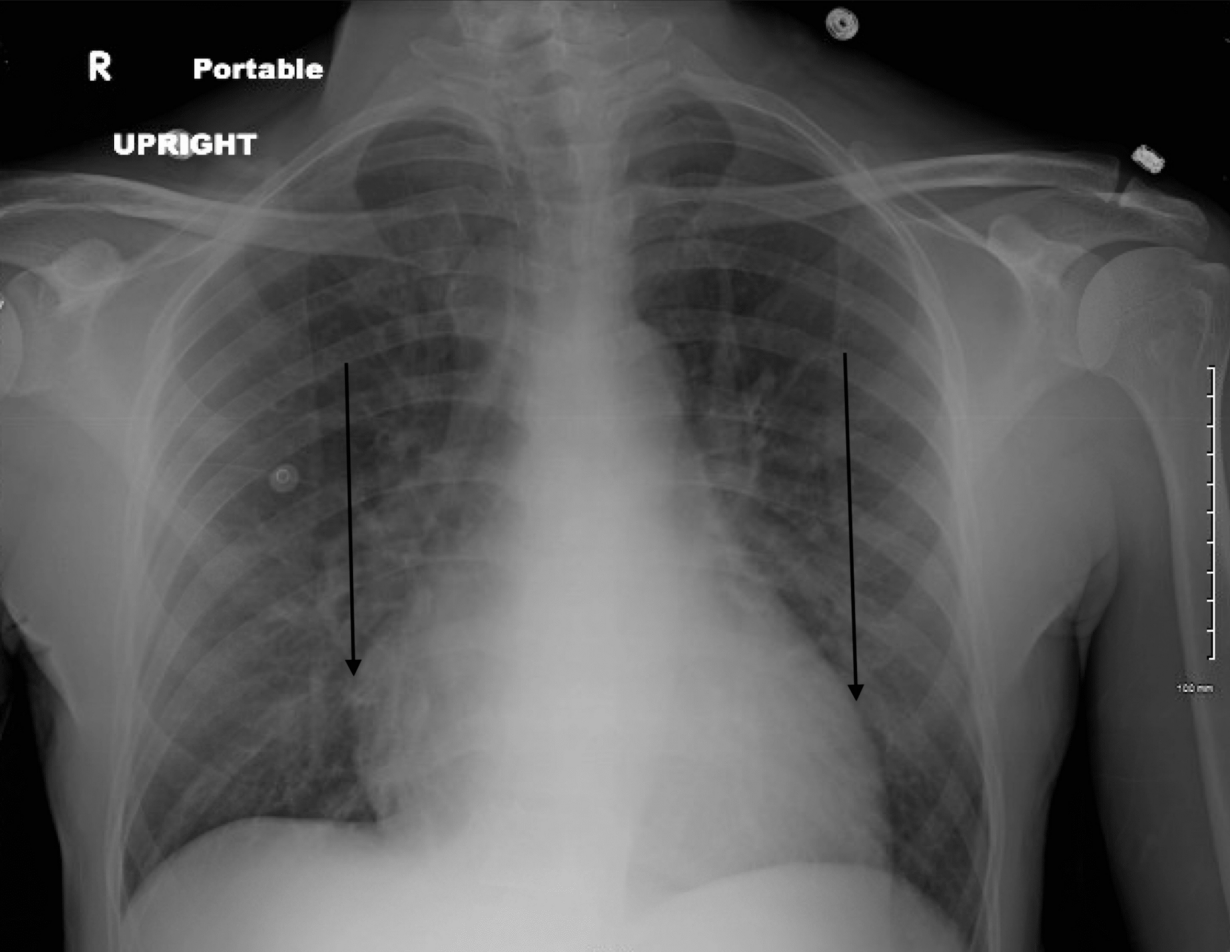
Upright X-ray showing mild cardiomegaly (black arrows) X-ray: X-radiation

**Figure 3:**
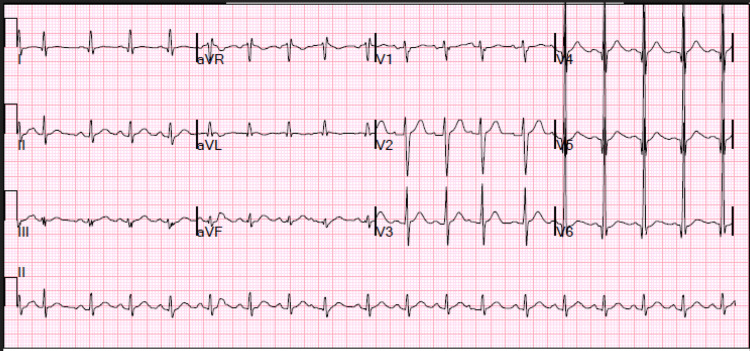
ECG on admission depicting tachycardia and ventricular hypertrophy in leads 4-6 ECG: electrocardiography

Significant initial laboratory results include the following: cross-linked dimeric degradation product (D-dimer), N-terminal pro-B-type natriuretic peptide (NT-proBNP), high sensitive cardiac troponin I, and creatine kinase-myocardial band (CK-MB) (Table 1). An elevated D-dimer prompted chest computed tomography pulmonary angiogram (CTPA) (Figure 1), which revealed no signs of pulmonary embolism, patchy ground glass opacities within the lingula and left lower lobe, mild dependent changes with subsegmental atelectasis within the bilateral lower lobes, and mildly enlarged main pulmonary artery, suggestive of underlying pulmonary artery hypertension, with an upright X-ray showing signs of cardiomegaly (Figure 2). Electrocardiography (ECG) revealed sinus tachycardia and left ventricular hypertrophy (LVH) (Figure 3). A two-dimensional (2D) echocardiogram showed an ejection fraction (EF) of 36%, with diffuse mild dilation of the atria and ventricles and trace mitral and tricuspid regurgitation (Video 1).

**Table 1:** Laboratory analysis NT-proBNP: N-terminal pro-B-type natriuretic peptide, CK-MB: creatine kinase-myocardial band, TSH: thyroid-stimulating hormone

Variables	On admission	Day 2	Day 3	Reference values
D-dimer (ug/mL)	1.07	–	–	<0.25
NT-proBNP (pg/mL)	5,485	–	–	
Sensitive cardiac troponin I (ng/mL)	20.9	–	–	<0.4
CK-MB (ng/mL)	1.8	–	–	0-4.9
TSH (uU/mL)	0.01	–	–	0.4-4.0
T3 (ng/dL)	–	459.37	–	100-300
Free T4 (ug/dL)	–	6.58	_	0.8-1.8
Thyroid-stimulating immunoglobulin (IU/L)	–	_	127	<1.75
Thyroglobulin antibody (IU/L)	–	_	<1.0	115-120
Thyroid peroxidase antibody (IU/mL)	–	_	281	<34-35
Thyroid-stimulating hormone receptor antibody (IU/L)	–	–	39.60	<1.75

**Video 1:** 2D Doppler echocardiogram showing an EF of 36% 2D: two dimensional, EF: ejection fraction

**Figure 4:**
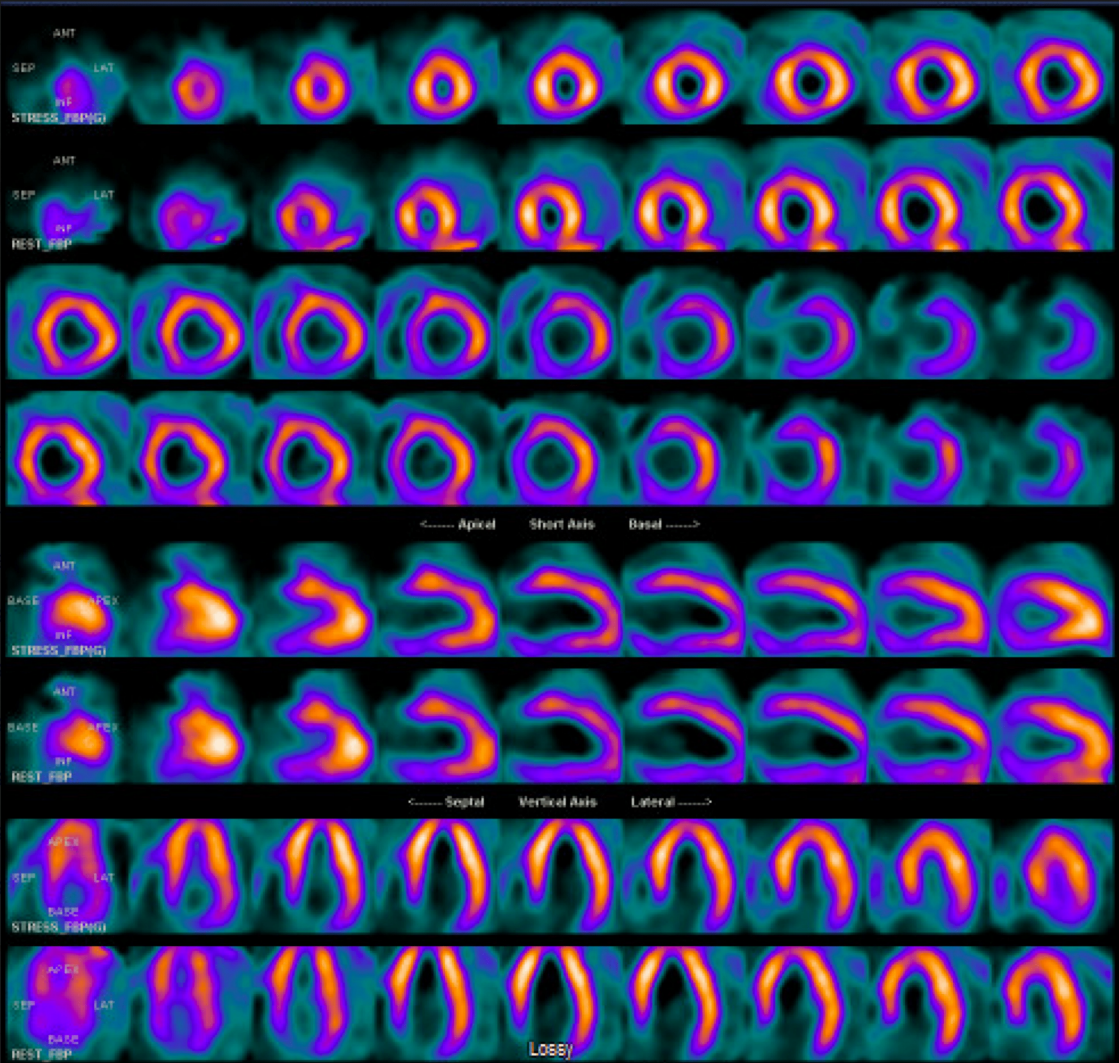
Nuclear medicine myocardial perfusion SPECT SPECT: single-photon emission computed tomography

On the second day of admission, the patient was placed in droplet isolation according to hospital policy due to COVID-19. It is important to note that the initial rapid at-home SARS-CoV-2 test was positive, but on hospital reassessment using PCR testing, the patient was found to be negative. Together with the lack of overt symptoms on physical examination, we suspect that this was likely a false-positive result. While SARS-CoV-2 has been associated with dilated cardiomyopathy in rare cases, we did not believe that there was sufficient evidence in this patient to include it as a likely differential diagnosis. His free T4 was 6.58 ug/dL and total T3 was 459.37 ng/dL, with a suppressed thyroid-stimulating hormone (TSH), indicating a hyperthyroid state. A thyroid ultrasound indicated an enlarged and relative hypervascular thyroid. However, due to the prior CTPA use of iodine, the radioactive iodine uptake study could not be completed. Nuclear medicine myocardial perfusion single-photon emission computed tomography (SPECT) at rest and stress indicated no evidence of a fixed defect or stress-induced ischemia (Figure 4).

The third day of admission resulted in serology studies, with thyroid-stimulating immunoglobulin of 127 IU/L, thyroglobulin antibody < 1.0 IU/mL, thyroid peroxidase antibody of 281 IU/mLco, and thyroid-stimulating hormone receptor antibody of 39.60 IU/L, indicating Grave’s disease. The treatments provided were as follows: methimazole dosing titrated to 20 mg tablet, orally, twice daily; metoprolol 50 mg tablet, orally, daily; and Entresto 1 tablet (49/51 mg), orally, twice daily. A summary of the patient’s notable laboratory values throughout the hospital stay is provided in Table 1.

The patient significantly improved. Six weeks later, he was clinically and biochemically euthyroid. His heart failure medication was discontinued, A repeat echocardiogram showed normal left ventricular (LV) and right ventricular (RV) systolic function with an ejection fraction of 56%.

## Discussion

Hyperthyroidism is a common metabolic disorder with a well-understood cardiovascular manifestation [17]. Congestive heart failure (CHF) was the initial clinical presentation in approximately 6% of patients with hyperthyroidism [16,18]. In hyperthyroidism, cardiac contractility and cardiac output are enhanced, and systemic vascular resistance is decreased, while in hypothyroidism, the opposite is true [19]. Taken together, this creates a hyperdynamic circulatory state [20,21]. While thyrotoxicosis has commonly been shown to contribute to the signs and symptoms of CHF [19,22], it has rarely been associated with the development of left ventricular systolic dysfunction [15,23,24]. Hyperthyroidism is a very rare (<1%) cause of dilated cardiomyopathy [25]. Of 6% of thyrotoxic individuals who develop symptoms of heart failure, less than 1% develop dilated cardiomyopathy with impaired left ventricular systolic function [16,18]. Siu et al. was the first systematic study to show that persistent and potentially fatal dilated cardiomyopathy developed in approximately 1% (6/519 patients) of patients with thyrotoxicosis, with up to one-third of patients with left ventricular systolic dysfunction at presentation maintaining persistent dilated cardiomyopathy during long-term follow-up [18]. Haidous et al. [14] did recently report a similar case of a patient who developed left ventricular dysfunction with an ejection fraction between 30% and 35%. Our study further adds evidence to the existence of significant left ventricular heart failure that may occur with untreated or inappropriately treated hyperthyroidism.

Treatment is centered around the management of cardiovascular complications, that is, control of heart rate and thyroid-specific therapy to restore a euthyroid state that will resolve the signs and symptoms of heart failure [16]. Umpierrez et al. [26] found that hyperthyroidism-induced ventricular dysfunction was completely reversible in 71% of patients included in the study, with significant improvement in the remaining 29%. Therefore, especially in a patient with severely reduced left ventricular function, it is imperative to identify the cause of heart failure promptly. Physicians must be aware of the significant cardiac effects that may arise in a hyperthyroidism patient with developing thyrotoxicosis.

## Conclusions

In conclusion, our study highlights the importance of assessing thyroid hormone status in patients who present with heart failure, as hyperthyroidism can be a rare but significant cause of dilated cardiomyopathy. The findings of our study further add evidence to the existence of significant left ventricular heart failure that may occur with untreated or inappropriately treated hyperthyroidism. Early recognition and management of thyrotoxicosis can reverse cardiovascular manifestations and prevent irreversible cardiomyopathy. However, it is essential to note that the reversibility of hyperthyroidism-induced ventricular dysfunction may vary, and further studies are needed to determine the optimal management strategies. Additionally, our study highlights the need for more research to better understand the underlying mechanisms and the long-term outcomes of hyperthyroidism-induced cardiomyopathy. In summary, physicians must be aware of the potential cardiac effects of hyperthyroidism and the importance of timely and appropriate management.
